# Prevalent Morphometrically Assessed Vertebral Fractures in Individuals With Type 2 Diabetes, Prediabetes and Normal Glucose Metabolism: The Maastricht Study

**DOI:** 10.3389/fendo.2022.832977

**Published:** 2022-02-18

**Authors:** Veerle van Hulten, Cindy Sarodnik, Johanna H. M. Driessen, Nicolaas C. Schaper, Piet P. M. M. Geusens, Carol A. B. Webers, Geert-Jan Dinant, Ramon P. G. Ottenheijm, Nicklas H. Rasmussen, Rikke Viggers, Coen D. A. Stehouwer, Carla J. H. van der Kallen, Miranda T. Schram, Sandrine P. G. Bours, Pieter C. Dagnelie, Joop P. van den Bergh

**Affiliations:** ^1^ Department of Clinical Pharmacy and Toxicology, Maastricht University Medical Centre+ (MUMC+), Maastricht, Netherlands; ^2^ School of Nutrition and Translational Research in Metabolism (NUTRIM), Maastricht University, Maastricht, Netherlands; ^3^ Cardiovascular Research Institute Maastricht (CARIM), Maastricht University, Maastricht, Netherlands; ^4^ Division of Rheumatology, Department of Internal Medicine, Maastricht University Medical Centre+ (MUMC+), Maastricht, Netherlands; ^5^ Department of Internal Medicine, Maastricht University Medical Centre+ (MUMC+), Maastricht, Netherlands; ^6^ Care and Public Health Research Institute (CAPHRI), Maastricht University, Maastricht, Netherlands; ^7^ Biomedical Research Institute, University Hasselt, Hasselt, Belgium; ^8^ University Eye Clinic Maastricht, Maastricht University Medical Centre+ (MUMC+), Maastricht, Netherlands; ^9^ Department of Family Medicine, Care and Public Health Research Institute (CAPHRI), Maastricht University, Maastricht, Netherlands; ^10^ Steno Diabetes Center North Jutland, Department of Endocrinology, Aalborg University Hospital, Aalborg, Denmark; ^11^ Department of Clinical Medicine, Aalborg University, Aalborg, Denmark; ^12^ Heart and Vascular Center, Maastricht University Medical Center+ (MUMC+), Maastricht, Netherlands; ^13^ Department of Epidemiology, Maastricht University, Maastricht, Netherlands; ^14^ Subdivision of Endocrinology, Department of Internal Medicine, VieCuri Medical Center, Venlo, Netherlands

**Keywords:** bone, type 2 diabetes, vertebral fracture (VF), vertebral fracture assessment (VFA), dual energy X-ray absorptiometry (DEXA)

## Abstract

**Background:**

Type 2 diabetes (T2D) is frequently reported to be associated with an increased fracture risk. Epidemiological data on prevalent morphometric vertebral fractures (VFs) in T2D are sparse and even less is known in the prediabetic state.

**Purpose:**

To determine the association between prevalence and severity of morphometric VFs and glucose metabolism state: normal glucose metabolism (NGM), impaired glucose metabolism (prediabetes) or T2D.

**Methods:**

This study included cross-sectional data from 3625 participants of the Maastricht Study who had a vertebral fracture assessment on lateral Dual Energy X-Ray Absorptiometry images. VFs were classified based on morphometric assessment into mild, moderate and severe VFs (respectively 20–24%, 25–39% or ≥40% reduction in expected vertebral body height). Logistic regression models were used to investigate the association between glucose metabolism status and the prevalence and severity of VFs. Analyses were adjusted for subject characteristics and life-style factors.

**Results:**

T2D individuals were older (62.8 ± 7.5 years old) and less often female (30.5%) compared to the NGM group (57.7 ± 8.5 years old, and 58.8% female, respectively). At least one mild, moderate or severe prevalent VF was found in 8.6% of the men and 2.2% of the women in the T2D group, in 9.4% and 8.4% in the prediabetes group and in 9.1% and 4.8% in the NGM group, respectively. After adjustment T2D in women was associated with a lower probability of having a prevalent VF compared to NGM [adjusted OR 0.25 (95% CI 0.09-0.65)], while this was not the case for prediabetes. Furthermore, women with T2D had a significantly lower probability of a prevalent moderate or severe VF [adjusted OR 0.32 (95% CI 0.11-0.96)]. In men there was no significant association between T2D or prediabetes and prevalent VFs.

**Conclusion:**

Women with T2D had a lower probability of prevalent VFs compared to women with a normal glucose metabolism, while this was not the case for men with T2D and participants with prediabetes.

## Introduction

Type 2 diabetes mellitus (T2D) is a chronic disease characterized by macro- and microvascular complications. The impact of diabetes on bone metabolism may lead to a deterioration of bone microarchitecture and lower bone strength. These alterations could be regarded as a skeletal complication of T2D which, in combination with increased risk of falling, may lead to an increased fracture risk (1). Interestingly, a higher risk of fractures has been reported in T2D despite a normal or even higher areal bone mineral density (aBMD) compared to non-diabetic individuals (2–4).

This increased fracture risk has largely been shown for hip and non-vertebral fractures (5–7), while literature on prevalent vertebral fractures (VFs) in T2D is sparse and inconclusive. In a recent combined meta-analysis of individual data obtained from cohort studies and previously published studies, a decreased risk of prevalent VFs in T2D compared to no diabetes was reported (8). However, it was reported that information of the type of treatment individuals with T2D were receiving and comorbidities was lacking and that there could have been bias due to loss to follow-up of participants in the individual data analysis. Furthermore, the ascertainment of prevalent VFs differed among included studies. Lastly, the possible difference in prevalent VF risk between men and women was not fully elucidated, since some studies included only men or only women.

It is, however, of great importance to identify prevalent VFs in individuals with clinical risk factors for fractures, since the presence of a prevalent VF is strongly associated with the risk of subsequent vertebral and non-vertebral fractures (9–11) and mortality risk (12, 13).

Because individuals with T2D tend to fracture at a higher BMD T-score compared to healthy individuals without diabetes, it has been suggested that individuals with T2D should be systematically assessed for the presence of a VF, preferably by the assessment of lateral spine images of modern dual energy X-ray absorptiometry (DXA) devices (14, 15) if there is an indication for BMD testing based on the clinical fracture risk profile.

In this study, we aimed to assess prevalent morphometric VFs using lateral DXA images in participants of the Maastricht Study, an extensive phenotyping study on determinants of type 2 diabetes, its complications, and its comorbidities (16). In addition, we aimed to compare the presence and severity of prevalent VFs between participants with normal glucose metabolism (NGM), prediabetes and T2D.

## Materials and Methods

### The Maastricht Study: Population and Design

We used data from The Maastricht Study, an observational prospective population-based cohort study. The rationale and methodology have been described previously (16). In brief, the study focuses on the etiology, pathophysiology, complications and comorbidities of T2D and is characterized by an extensive phenotyping approach. Eligible for participation were all individuals aged between 40 and 75 years and living in the southern part of the Netherlands. Participants were recruited through mass media campaigns and from the municipal registries and the regional Diabetes Patient Registry *via* mailings. Recruitment was stratified according to known T2D status, with an oversampling of individuals with T2D, for reasons of efficiency. The present report includes cross-sectional data from the first 7689 participants, who completed the baseline survey between November 2010 and December 2017. The examinations of each participant were performed within a time window of three months. The study has been approved by the institutional medical ethical committee (NL31329.068.10) and the Minister of Health, Welfare and Sports of the Netherlands (Permit 131088-105234-PG). All participants gave written informed consent.

To determine glucose metabolism status, all participants, except those who used insulin, underwent a standardized 2-h 75g oral glucose tolerance test (OGTT) after an overnight fast. For safety reasons, participants with a fasting glucose level above 11.0 mmol/L, as determined by a finger prick, did not undergo the OGTT. For these individuals (n=13), fasting glucose level and information about diabetes medication were used to determine glucose metabolism status. Glucose metabolism status was defined according to the WHO 2006 criteria into normal glucose tolerance (NGT), impaired fasting glucose (fasting plasma glucose (FPG) 6.1-6.9 mmol/l and 2h plasma glucose <7.8 mmol/l) or impaired glucose tolerance (FPG <7.0 mmol/l and 2h plasma glucose 7.8 - 11.1 mmol/l), which were both considered as prediabetes, and T2D (FPG ≥7.0 mmol/l or 2h plasma glucose ≥11.1mmol/l) (17). Individuals without type 1 diabetes on diabetes medication were classified as having T2D (16).

### Methods

The participants were invited for a DXA in the period of October 2016 until July 2019. In this cross-sectional study we used data from all participants of The Maastricht Study who had a BMD measurement at the lumbar spine and hip and lateral spine imaging by DXA (Hologic QDR 4500, Hologic, Bedford, MA, USA). BMD measurements were classified according to the lowest value of T-score in the total hip/femoral neck or lumbar spine: osteoporosis as T-score ≤−2.5, osteopenia as T-score between −2.5 and −1.0, and normal BMD as T-score ≥−1.0.

The densitometric lateral spine images were used for vertebral fracture assessment (VFA). The BMD measurements and lateral spine images were performed in a clinical facility and evaluated by experienced radiologists, who were blinded with reference to the glucose metabolism status of the participants, using a standardized land marking protocol. First, the image quality was evaluated, and the vertebrae were labeled, starting with the identification of the fourth lumbar vertebra. Subsequently, the evaluable vertebrae were determined. A vertebra was considered evaluable if the posterior and anterior cortices and both endplates were fully and clearly visible. If this was not the case, the vertebra was not evaluated. The standardized land marking protocol uses 95 points to represent the circumferential vertebral borders, including right/left/central endplate margins and anterior/posterior margins.

For the purpose of this study, the vertebral shape and the appearance of the end plate were evaluated to differentiate between VFs and vertebrae with other deformities, e.g. degenerative changes or Scheuermann’s disease, by two trained clinicians (CS, SB) who were blinded with reference to the glucose metabolism status of the participants. Subsequently, VFs were graded by morphometric assessment according to Genant et al. (18) as grade 0, <20% reduction in expected vertebral body height at the anterior, mid, or posterior location; grade 1, 20–24% (mild VF); grade 2, 25–39% (moderate VF); or grade 3, ≥40% (severe VF) reduction, respectively. Patients were classified according to the most severe VF as those without VFs, those with at least one mild VF, or those with at least one moderate or severe VF.

### Covariates

For the current study age, sex, smoking status, BMD, body mass index (BMI), educational level, and time gap (time in months between baseline visit and DXA scan) were all considered as potential confounders. BMI was calculated by dividing weight in kilogram by height in meters squared, which were measured while wearing light clothing without shoes, using a scale and stadiometer to the nearest 0.1 cm and 0.5 kg, respectively. Smoking status, fracture history, and educational level were assessed using a questionnaire. Smoking status was categorized into never, former and current, and educational level was categorized into low, medium and high.

### Statistical Analyses

General characteristics and the presence, severity and number of prevalent VFs were calculated for all three groups, being NGM, prediabetes and T2D, separately. This data was additionally stratified for men and women, due to the skewed distribution of men and women per group. Categorical variables are presented as number of participants (%) and continuous variables are presented as mean values [standard deviation (SD)].

Logistic regression was used to investigate the association between glucose metabolism status and the presence of prevalent VFs, using both crude and adjusted models. To test for interaction between glucose metabolism status and sex, glucose metabolism status and BMI, and glucose metabolism status and BMD, interaction terms (e.g. dummy-coded glucose metabolism status variables * sex, etc.) were incorporated into the logistic regression models. A P_interaction_<0.10 was considered to be statistically significant, as is common in statistical interaction testing (19). The main analysis investigated the association between glucose metabolism status and the presence of at least one prevalent VF. Furthermore, the relationship between glucose metabolism status and moderate and severe VFs versus no VFs was studied. Both analyses were stratified by sex. The results from these analyses are presented as odds ratios (OR), with 95% confidence intervals (CI). For glucose metabolism status, NGM was set as the reference group.

Additionally, a logistic regression analysis was performed within the T2D cohort to study the association between diabetes related characteristics [glycated hemoglobin A1c (HbA1c), diabetes duration and the presence of microvascular complications (MVCs)] and the presence of prevalent VFs.

Multiple models were created for the logistic regression analyses, providing ORs adjusted for the potential confounders depending on the number of events per analysis. For every ten events, meaning cases of a VF, one potential confounder was added to the model.

Lastly, a sensitivity analysis was performed including only participants with screen-detected T2D (T2D detected in the Maastricht Study by OGTT) from the study population.

All analyses were performed with IBM Statistical Package for Social Sciences for Macintosh, version 25.0 (IBM SPSS, IBM Corp, Armonk, NY, USA). *P*-values<0.05 were considered statistically significant.

## Results

The general characteristics of the study population are summarized in **Table 1**. A total of 3,626 participants were included in this study (2346 NGM (64.7%), 546 prediabetes (15.1%) and 734 T2D (20.2%)) (**Supplemental Figure 1**). The mean age was 59 ± 8.5 years and 1,853 (51.1%) were female. Since a significant interaction effect (*P*-value = 0.012) between sex and glucose metabolism status was found, all results are presented separately for men and women. In men (n=1,773), 966 (54.4%) had NGM, 297 (16.8%) prediabetes and 510 (28.8%) T2D. In women (n=1,853), 1380 (74.5%) had NGM, 249 (13.4%) prediabetes and 224 (12.1%) T2D.

**Table 1:** General characteristics of the study population.

	Men (N = 1773)			Women (N = 1853)		
	NGM (n = 966)	Prediabetes (n = 297)	T2D (n = 510)	NGM (n = 1380)	Prediabetes (n = 249)	T2D (n = 224)
Age (years)	58.7 (8.4)	61.8 (7.6)	63.3 (7.2)	57.1 (8.5)	60.6 (8.7)	61.5 (7.9)
BMI (kg/m^2^)	26.1 (3.2)	27.8 (3.4)	29.3 (4.2)	25.0 (3.8)	27.7 (4.7)	29.7 (5.0)
**Educational level**						
Low	226 (23.4%)	92 (31.0%)	194 (38.0%)	434 (31.4%)	111 (44.6%)	133 (59.4%)
Medium	263 (27.2%)	73 (24.6%)	138 (27.1%)	402 (29.1%)	67 (26.9%)	57 (25.4%)
High	477 (49.4%)	132 (44.4%)	178 (34.9%)	544 (39.4%)	71 (28.5%)	34 (15.2%)
**Smoking status**						
Never	413 (42.8%)	90 (30.3%)	144 (28.2%)	592 (42.9%)	91 (36.5%)	90 (40.2%)
Former	431 (44.6%)	179 (60.3%)	301 (59.0%)	628 (45.5%)	129 (51.8%)	105 (46.9%)
Current	122 (12.6%)	28 (9.4%)	65 (12.7%)	160 (11.6%)	29 (11.6%)	29 (12.9%)
**Alcohol use**						
None	77 (8.0%)	22 (7.4%)	92 (18.0%)	254 (18.4%)	64 (25.7%)	104 (46.4%)
Low	670 (69.4%)	197 (66.3%)	317 (62.2%)	760 (55.1%)	120 (48.2%)	93 (41.5%)
High	219 (22.7%)	78 (26.3%)	101 (19.8%)	366 (26.5%)	65 (26.1%)	27 (12.1%)
History of CVD	110 (11.4%)	50 (16.8%)	153 (30.0%)	160 (11.6%)	35 (14.4%)	39 (17.4%)
History of fractures	414 (42.9%)	121 (40.7%)	178 (34.9%)	455 (33.0%)	82 (32.9%)	64 (28.6%)
Family history of fractures	397 (41.1%)	106 (35.7%)	183 (35.9%)	768 (55.7%)	125 (50.2%)	102 (45.5%)
Family history of osteoporosis	57 (5.9%)	17 (5.7%)	33 (6.5%)	246 (17.8%)	42 (16.9%)	36 (16.1%)
**Medication use**						
Antihyperglycemic drugs	0 (0%)	0 (0%)	377 (73.9%)	0 (0%)	0 (0%)	153 (68.3%)
Insulin	0 (0%)	0 (0%)	95 (18.6%)	0 (0%)	0 (0%)	38 (17.0%)
Oral antihyperglycemic drugs	0 (0%)	0 (0%)	354 (69.4%)	0 (0%)	0 (0%)	138 (61.6%)
Blood pressure lowering drugs	250 (25.9%)	129 (43.4%)	366 (71.8%)	240 (17.4%)	109 (44.0%)	153 (68.3%)
Psychoactive drugs	23 (2.4%)	6 (2.0%)	17 (3.3%)	77 (5.6%)	20 (8.1%)	24 (10.7%)
Anti-osteoporosis treatment	4 (0.4%)	2 (0.7%)	2 (0.4%)	29 (2.1%)	7 (2.8%)	3 (1.3%)
Glucocorticoids	3 (0.3%)	2 (0.7%)	6 (1.2%)	4 (0.3%)	1 (0.4%)	6 (2.7%)
**Diabetes-related characteristics**						
HbA1c (mmol/mol)	35.1 (3.9)	37.6 (4.3)	51.1 (11.4)	35.1 (3.8)	37.6 (4.4)	50.5 (11.8)
HbA1c (%)	5.4 (0.4)	5.6 (0.4)	6.8 (1.0)	5.4 (0.4)	5.6 (0.4)	6.8 (1.1)
Diabetes duration at inclusion (years)	0 (0)	0 (0)	7.0 (7.4)	0 (0)	0 (0)	5.5 (6.4)
Retinopathy	2 (0.2%)	6 (2.0%)	21 (4.1%)	7 (0.5%)	1 (0.4%)	5 (2.2%)
Impaired vibration sensation	57 (5.9%)	32 (10.8%)	83 (16.3%)	52 (3.8%)	17 (6.8%)	27 (12.1%)
Nephropathy^a^	107 (11.1%)	44 (14.8%)	139 (27.3%)	149 (10.8%)	38 (15.3%)	42 (18.8%)
eGFR <60ml/min	50 (5.2%)	29 (9.8%)	51 (10.0%)	124 (9.0%)	25 (10.1%)	32 (14.3%)
Albuminuria >30mg/24h	68 (7.0%)	17 (5.7%)	103 (20.2%)	30 (2.2%)	14 (5.6%)	17 (7.6%)
BMD LS (g/cm^2^)	1.09 (0.18)	1.14 (0.19)	1.19 (0.20)	0.97 (0.16)	1.00 (0.17)	1.05 (0.19)
BMD TH (g/cm^2^)	0.98 (0.13)	1.01 (0.13)	1.03 (0.16)	0.84 (0.12)	0.86 (0.13)	0.90 (0.14)
BMD FN (g/cm^2^)	0.80 (0.12)	0.82 (0.12)	0.84 (0.17)	0.72 (0.11)	0.73 (0.11)	0.75 (0.13)
Normal BMD	430 (44.5%)	136 (45.8%)	261 (51.2%)	388 (28.1%)	78 (31.3%)	100 (44.6%)
Osteopenia	472 (48.9%)	146 (49.2%)	234 (45.9%)	703 (50.9%)	129 (51.8%)	98 (43.8%)
Osteoporosis	64 (6.6%)	15 (5.1%)	15 (2.9%)	289 (20.9%)	42 (16.9%)	26 (11.6%)

Values show mean (SD) or number (%).

VF, vertebral fracture; NGM, normal glucose metabolism; T2D, type 2 diabetes; eGFR, estimated glomerular filtration rate; BMD, bone mineral density; LS, lumbar spine; TH, total hip; FN, femoral neck; VFA, vertebral fracture assessment; CVD, cardiovascular disease; BMI, body mass index.

a defined as an estimated glomerular filtration rate (eGFR) below 60 ml/min/1.73 m2, albuminuria, or both.

**Table 2** shows the number, severity and location of prevalent VFs per glucose metabolism status group. At least one mild, moderate or severe prevalent VF was found in 8.6% of the men and 2.2% of the women in the T2D group, in 9.4% and 8.4% in the prediabetes group and in 9.1% and 4.8% in the NGM group, respectively.

**Table 2:** Number, severity and location of prevalent vertebral fractures in men and women with normal glucose metabolism, prediabetes and type 2 diabetes.

	Men (N = 1773)			Women (N = 1853)		
	NGM (n = 966)	Prediabetes (n = 297)	T2D (n = 510)	NGM (n = 1380)	Prediabetes (n = 249)	T2D (n = 224)
**Presence of VFs**						
**By number of VFs**						
No VF	878 (90.9%)	269 (90.6%)	466 (91.4%)	1314 (95.2%)	228 (91.6%)	219 (97.8%)
≥ 1 VF	88 (9.1%)	28 (9.4%)	44 (8.6%)	66 (4.8%)	21 (8.4%)	5 (2.2%)
1 VF	61 (6.3%)	23 (7.7%)	36 (7.1%)	58 (4.2%)	15 (6.0%)	5 (2.2%)
2 VFs	20 (2.1%)	4 (1.3%)	8 (1.6%)	5 (0.4%)	4 (1.6%)	0 (0%)
3 VFs	7 (0.7%)	0 (0%)	0 (0%)	1 (0.1%)	2 (0.8%)	0 (0%)
> 3 VFs	0 (0%)	1 (0.3%)	0 (0%)	2 (0.1%)	0 (0%)	0 (0%)
**By severity**						
Mild VF	39 (4.0%)	8 (2.7%)	17 (3.3%)	24 (1.7%)	8 (3.2%)	1 (0.4%)
Moderate VF	43 (4.5%)	18 (6.1%)	22 (4.3%)	37 (2.7%)	10 (4.0%)	4 (1.8%)
Severe VF	6 (0.6%)	2 (0.7%)	5 (1.0%)	5 (0.4%)	3 (1.2%)	0 (0%)
**By location**						
Thoracic VF	66 (6.8%)	19 (6.4%)	36 (7.1%)	49 (3.6%)	15 (6.0%)	4 (1.8%)
Lumbar VF	33 (3.4%)	11 (3.7%)	11 (2.2%)	23 (1.7%)	8 (3.2%)	1 (0.4%)

Values show mean (SD) or number (%).

VF, vertebral fracture; NGM, normal glucose metabolism; T2D, type 2 diabetes.

In women, T2D was associated with a lower probability of having at least one prevalent VF compared to NGM [adjusted OR 0.25 (95% CI 0.09-0.65)], while this was not the case for prediabetes (**Table 3**).

**Table 3:** Odds of prevalent vertebral fractures in prediabetes and type 2 diabetes, stratified by sex.

Men (N = 1773)	All VFs (N = 160)	No VF (N = 1613)	Crude OR (95% CI)	Adjusted OR* (95% CI)
NGM (n=966)	88 (9.1%)	878 (90.9%)	Reference	Reference
Prediabetes (n=297)	28 (9.4%)	269 (90.6%)	1.04 (0.66-1.62)	0.99 (0.63-1.58)
T2D (n=510)	44 (8.6%)	466 (91.4%)	0.94 (0.65-1.38)	0.87 (0.57-1.34)
**Women (N = 1853)**	**All VFs (N = 92)**	**No VF (N = 1761)**	**Crude OR (95% CI)**	Adjusted OR* (95% CI)
NGM (n=1380)	66 (4.8%)	1314 (95.2%)	Reference	Reference
Prediabetes (n=249)	21 (8.4%)	227 (91.6%)	1.83 (1.10-3.06)	1.22 (0.70-2.11)
T2D (n=224)	5 (2.2%)	219 (97.8%)	0.46 (0.18-1.14)	0.25 (0.09-0.65)

Values show numbers (%).

*Logistic regression analysis, adjusted for age, smoking status, bone mineral density, body mass index, educational level, and time gap (time in months between baseline visit and DXA scan).

VF, vertebral fracture; NGM, normal glucose metabolism; T2D, type 2 diabetes; OR, odds ratio; CI, confidence interval.

Furthermore, T2D was associated with a significantly lower probability of a moderate or severe VF [adjusted OR 0.32 (95% CI 0.11-0.96)] (**Table 4**). In men there was no significant association between T2D or prediabetes and prevalent VFs (**Tables 3**, **4**).

**Table 4:** Odds of prevalent moderate and severe vertebral fractures in prediabetes and type 2 diabetes, stratified by sex.

Men (N = 1709)	Moderate or severe VF (N = 94)	No VF (N = 1613)	Crude OR (95% CI)	Adjusted OR* (95% CI)
NGM (n=927)	49 (5.3%)	878 (94.7%)	Reference	Reference
Prediabetes (n=289)	20 (6.9%)	269 (93.1%)	1.33 (0.78-2.28)	1.26 (0.72-2.21)
T2D (n=493)	27 (5.5%)	466 (94.5%)	1.04 (0.64-1.67)	1.01 (0.59-1.72)
**Women (N = 1820)**	**Moderate or severe VF (N = 59)**	**No VF (N = 1760)**	**Crude OR (95% CI)**	Adjusted OR* (95% CI)
NGM (n=1356)	42 (3.1%)	1314 (96.9%)	Reference	Reference
Prediabetes (n=241)	13 (5.4%)	228 (94.6%)	1.78 (0.94-3.38)	1.15 (0.58-2.27)
T2D (n=223)	4 (1.8%)	219 (98.2%)	0.57 (0.20-1.61)	0.32 (0.11-0.96)

*Logistic regression analysis, adjusted for age, smoking status, bone mineral density, and body mass index.

VF, vertebral fracture; NGM, normal glucose metabolism; T2D, type 2 diabetes; OR, odds ratio; CI, confidence interval.

In men with T2D, we did not find a significant association between HbA1c, diabetes duration or the presence of MVCs with the presence of a prevalent VF. In women, the number of participants with T2D and a VF (n=5 (2.2%)) was too low for further analysis.

The results from the sensitivity analysis, including only screen-detected T2D, did not show any significant results. Neither men nor women with screen-detected T2D had a significantly altered probability of prevalent VFs compared to participants in the NGM group (**Supplemental Table 1**). Likewise, the probability of prevalent moderate or severe VFs was not significantly associated with the presence of screen-detected T2D, in neither men nor women (**Supplemental Table 2**).

## Discussion

In this study, we aimed to investigate the association between glucose metabolism status and the presence and severity of prevalent morphometrically identified VFs on lateral DXA images. Women with T2D had a lower probability of prevalent VFs compared to women with a normal glucose metabolism, while this was not the case for men with T2D and participants with prediabetes.

Our results are in line with the sensitivity analysis of the meta-analysis by Koromani et al. (8), which reported a lower odds of prevalent VFs with T2D in men and women (OR: 0.84; 95% CI: 0.74 – 0.95) based on individual participant data (IPD) of five cohorts (20–24), and two published studies (25, 26). In this sensitivity analysis 2 studies causing heterogeneity were excluded from the meta-analysis. For one study, reason for exclusion was that participants were recruited from a tertiary center, and consequently participants had an abnormally low BMD (T-score of -2.0 for women, -2.4 for men). The other excluded study relied on a national database for the ascertainment of VFs, which possibly causes T2D to be diagnosed with VFs more often, since they are usually under stricter supervision. As in our study, the included studies reported that the OR for VFs was significantly lower among women but not among men.

Our study confirms the results of the meta-analysis by Koromani et al. (8), and provides additional certainty due to the comprehensive design of The Maastricht Study. Firstly, in the cohorts and studies analyzed by Koromani et al. (8), VFs were assessed on lateral radiographs, except for one cohort using lateral DXA images (22), and only moderate and severe VFs were included. In our study mild, moderate and severe VFs were assessed on lateral DXA images. Furthermore, only in two of the five meta-analysis cohorts, women and men were studied (20, 23). However, in the other three cohorts and in one published study only women (21, 22, 24, 26) were included, and in one study only men were included (25). Additionally, some studies included in the meta-analysis by Koromani et al. (8) relied on GP records or self-reported T2D diagnosis (20, 21, 25, 26), which may lower the validity of diabetes classification. In our study, glucose metabolism status was independently investigated using the seven-point OGTT, providing accurate data on glucose metabolism status of all participants. Lastly, in two studies included in the meta-analysis, ORs were either not adjusted or only adjusted for a limited number of confounders (25, 26). In the Maastricht Study cohort, all participating men and women were extensively phenotyped, allowing us to accurately study men and women separately and to adjust our results adequately for potential confounders.

Based on the findings of our study in combination with individual participant data analyses reported by Koromani et al. (8) women with T2D have a lower probability of moderate and severe VFs and likely also of mild prevalent VFs while this is not the case for men. The underlying mechanism of this finding is not yet fully elucidated, given the limited number of studies, but several factors could play a role. Firstly, it could be speculated that the lower probability of prevalent VFs in women with T2D may be related to higher estrogen levels in women with T2D compared to women without T2D. Women with T2D generally have a higher BMI compared to women with NGM, as was the case in our cohort. In the postmenopausal state, there is a linear relationship between a higher BMI and higher free estrogen levels (27, 28). Furthermore, higher levels of estradiol were reported to be independently associated with the risk of T2D, after adjustment for BMI, glucose and insulin levels (29). Estrogen levels are known to be inversely proportional to fracture risk in postmenopausal women, since estrogen deficiency stimulates bone resorption and does not allow adequate bone formation (30). Thus, since most of the women with T2D included in our study were postmenopausal, they were likely to have higher estrogen levels compared to the women with NGM related to a higher body fat mass, which could contribute to the lower probability of prevalent VFs (31). This may especially be the case in the women with T2D in our cohort, who are characterized by a well-regulated T2D (mean HbA1C 50.5 mmol/mol) and by a relatively short time since the diagnosis of T2D (mean 5.5 years) so that possible detrimental effects of longer T2D duration on bone may not be present yet. This notion would be consistent with our sensitivity analysis which shows that the lower odds of prevalent VFs in T2D women was not found in screen-detected women with T2D, who had a lower BMI (28.7 ± 4.7 kg/m^2^) compared to women with previously diagnosed T2D (30.1 ± 5.1 kg/m^2^). The finding that the OR for VFs was lower in women with T2D even after adjustment for BMI could be explained by the fact that higher estrogen levels are correlated to fat mass distribution, which is not completely accounted for by adjustment for BMI.

Another potential explanation may be that postmenopausal women with T2D with a relatively short and well-regulated T2D were reported to have a better trabecular bone quality compared to women without T2D represented by a greater plate-like and less rod-like trabecular network (32). Since vertebrae primarily consist of trabecular bone a better trabecular bone quality could result in a lower probability of prevalent VFs.

In the meta-analysis of Koromani et al. (8) the lower risk of prevalent VFs was mostly present among the elderly and obese individuals. Unfortunately, due to the limited number of women with VFs, we were not able to stratify our analyses for BMI or BMD categories or to perform further in-depth analyses in the T2D cohort.

It is noteworthy that although the association between glucose metabolism status and the presence of prevalent morphometric VFs was only significant in women, the proportion of men with a prevalent VF was higher than in women (9.1% and 8.6% in men with NGM and T2D, respectively versus 4.8% and 2.2% in women). This was also shown in a study by Waterloo et al. (33), who hypothesized that this could be related to the lifestyle of men in their younger years since high-energy trauma was reported to be the cause of clinical VFs twice as often in men compared to women (34).

The present study has some limitations. Firstly, this study has a cross-sectional design. A causal relationship between glucose metabolism status and the risk of prevalent VFs could therefore not be studied. Additionally, for some participants the baseline visit took place several years before the DXA was performed, meaning we might not have a clear picture of the level of diabetes management at the time of the DXA. Furthermore, prevalent VFs were assessed by VFA on lateral DXA images, while the golden standard is radiography. As previously reported, the sensitivity of diagnosing VFs on DXA images is lower compared to X-ray, which could have led to an underestimation of prevalent VFs in our study (35). It is however unlikely that this explains the lower probability of VFs in women with T2D compared to NGM. In addition, a benefit of a VFA on DXA images is that no additional imaging was required for VF diagnosis and that DXA exposes individuals to a lower dose of radiation compared to X-ray. Moreover, it has been suggested that studies investigating VF prevalence in T2D could be biased by survivorship selection bias, resulting in a lower VF prevalence due to higher mortality in individuals with T2D compared to individuals without T2D (8). However, we believe that this is not an explanation for the lower probability of VFs in women with T2D in our study since the participants included in The Maastricht Study are thought to be relatively healthy compared to the average T2D individuals due to a participation bias (36, 37). Next, our results suggest a drastically lower OR for VFs in women with T2D (adjusted OR 0.25 (95% CI 0.09-0.65) compared to the sensitivity analysis by Koromani et al. (8) (OR: 0.84; 95% CI: 0.74 – 0.95), perhaps hinting at the possibility of unknown confounders for which we were not able to adjust. Regarding the diagnosis of T2D, we applied the WHO criteria by using a two-hour OGTT and we did not use HbA1C. This may be of influence to the number of participants classified as having T2D. However, HbA1C was reported to be insensitive for screening, especially with regard to undiagnosed diabetes and pre-diabetes (38). Lastly, due to the low proportion of VFs in T2D women, we were unable to perform in-depth analyses to shed light on the mechanism underlying our findings.

To conclude, we have found that women with T2D had a lower probability of prevalent VFs compared to women with a normal glucose metabolism, while this was not the case for men with T2D or for men and women with prediabetes.

## Data Availability Statement

The datasets presented in this article are not readily available because the dataset used in the present study was derived from the Maastricht Study. Upon reasonable request and with permission of the Maastricht Study management team, this dataset is available from the corresponding author. Requests to access the datasets should be directed to JB, jvdbergh@viecuri.nl.

## Ethics Statement

The studies involving human participants were reviewed and approved by Medical Ethical Committee (NL31329.068.10) and the Minister of Health, Welfare and Sports of the Netherlands (Permit 131088-105234-PG). The patients/participants provided their written informed consent to participate in this study.

## Author Contributions

Conceptualization: VH, CSa, JD, and JB. Methodology: VH, CSa, JD, and JB. Validation: VH, JD, and JB. Formal analysis: VH and JD. Investigation: staff of the Maastricht Study. Resources: Maastricht Study management team. Data curation: VH, JD, and Maastricht Study management team. Writing—original draft: VH. Writing—review and editing: VH, CS, JD, NS, PG, CW, G-JD, RO, NR, RV, CSt, CK, MS, SB, and JB. Visualization: VH and JD. Supervision: JB. Project administration: Maastricht Study management team. Funding acquisition: JD and JB. All authors contributed to the article and approved the submitted version.

## Funding

This study was supported by the European Regional Development Fund *via* OP-Zuid, the Province of Limburg, the Dutch Ministry of Economic Affairs (grant 31O.041), Stichting De Weijerhorst (Maastricht, the Netherlands), the Pearl String Initiative Diabetes (Amsterdam, the Netherlands), the Cardiovascular Center (CVC, Maastricht, the Netherlands), CARIM School for Cardiovascular Diseases (Maastricht, the Netherlands), CAPHRI School for Public Health and Primary Care (Maastricht, the Netherlands), NUTRIM School for Nutrition and Translational Research in Metabolism (Maastricht, the Netherlands), Stichting Annadal (Maastricht, the Netherlands), Health Foundation Limburg (Maastricht, the Netherlands), Perimed (Järfälla, Sweden), and by unrestricted grants from Janssen-Cilag B.V. (Tilburg, the Netherlands), Novo Nordisk Farma B.V. (Alphen aan den Rijn, the Netherlands), and Sanofi-Aventis Netherlands B.V. (Gouda, the Netherlands). VH received research funding from Novo Nordisk (Steno Collaborative Grant 2018).

## Conflict of Interest

The authors declare that the research was conducted in the absence of any commercial or financial relationships that could be construed as a potential conflict of interest.

## Publisher’s Note

All claims expressed in this article are solely those of the authors and do not necessarily represent those of their affiliated organizations, or those of the publisher, the editors and the reviewers. Any product that may be evaluated in this article, or claim that may be made by its manufacturer, is not guaranteed or endorsed by the publisher.

## Abbreviations

aBMD, Areal bone mineral density; BMI, Body mass index; CI, Confidence intervals; DXA, Dual energy X-ray absorptiometry; eGFR, Estimated glomerular filtration rate; FPG, Fasting plasma glucose; HbA1c, Glycated hemoglobin A1c; MVC, Microvascular complication; NGM, Normal glucose metabolism; NGT, Normal glucose tolerance; OR, Odds ratio; OGTT, Oral glucose tolerance test; T2D, Type 2 diabetes mellitus; VF, Vertebral fracture.
