# Supplementary material for: Prevalent Morphometrically Assessed Vertebral Fractures in Individuals With Type 2 Diabetes, Prediabetes and Normal Glucose Metabolism: The Maastricht Study
Source: Front Endocrinol (Lausanne). 2022 Feb 18;13:832977. doi: 10.3389/fendo.2022.832977 (PMC8894595; doi:10.3389/fendo.2022.832977)
Supplement: Supplementary file 1 [file DataSheet_1.pdf]

Supplementary Material

1 Supplementary Tables

Supplemental Table 1. Logistic regression analysis on the probability of prevalent vertebral fractures in prediabetes and screen-detected type 2 diabetes.

**Men** (N = 1350)	**Any VF** (N = 126)	**No VF** (N = 1224)	**Crude OR (95% CI)**	**Adjusted OR* (95% CI)**
**NGM** (n=966)	88 (9.1%)	878 (90.9%)	Reference	Reference
**Prediabetes** (n=297)	28 (9.4%)	269 (90.6%)	1.04 (0.66-1.62)	0.99 (0.62-1.58)
**T2Dscreen** (n=87)	10 (11.5%)	77 (88.5%)	1.30 (0.65-2.60)	1.21 (0.58-2.53)
**Women** (N = 1680)	**Any VF** (N = 89)	**No VF** (N = 1591)	**Crude OR (95% CI)**	**Adjusted OR* (95% CI)**
**NGM** (n=1380)	66 (4.8%)	1314 (95.2%)	Reference	Reference
**Prediabetes** (n=249)	21 (8.4%)	228 (91.6%)	1.83 (1.10-3.06)	1.25 (0.72-2.16)
**T2Dscreen** (n=51)	2 (3.9%)	49 (96.1%)	0.81 (0.19-3.41)	0.66 (0.15-2.85)

** Adjusted for age, smoking status, bone mineral density, body mass index, educational level, and time gap (time in months between baseline visit and DXA scan).*
VF, vertebral fracture; NGM, normal glucose metabolism; T2D, type 2 diabetes mellitus; OR, odds ratio; CI, confidence interval.

Supplemental Table 2. Logistic regression analysis on the probability of prevalent moderate and severe vertebral fractures, in prediabetes and screen-detected type 2 diabetes.

**Men** (N = 1302)	**Moderate or severe VF** (N = 78)	**No VF** (N = 1224)	**Crude OR (95% CI)**	**Adjusted OR* (95% CI)**
**NGM** (n=927)	49 (5.3%)	878 (94.7%)	Reference	Reference
**Prediabetes** (n=289)	20 (6.9%)	269 (93.1%)	1.33 (0.79-2.28)	1.28 (0.73-2.25)
**T2Dscreen** (n=86)	9 (10.5%)	77 (89.5%)	2.09 (0.99-4.43)	2.19 (0.97-4.95)
**Women** (N = 1648)	**Moderate or severe VF** (N = 57)	**No VF** (N = 1591)	**Crude OR (95% CI)**	**Adjusted OR* (95% CI)**
**NGM** (n=1356)	42 (3.1%)	1314 (96.9%)	Reference	Reference
**Prediabetes** (n=241)	13 (5.4%)	228 (94.6%)	1.78 (0.94-3.38)	1.15 (0.58-2.29)
**T2Dscreen** (n=51)	2 (3.9%)	49 (96.1%)	1.28 (0.30-5.43)	0.96 (0.22-4.22)

** Adjusted for age, smoking status, bone mineral density, and body mass index.*
VF, vertebral fracture; NGM, normal glucose metabolism; T2Dscreen, screen-detected type 2 diabetes mellitus; OR, odds ratio; CI, confidence interval.

2 Supplementary Figures

Supplemental Figure 1. Flowchart of the inclusion process

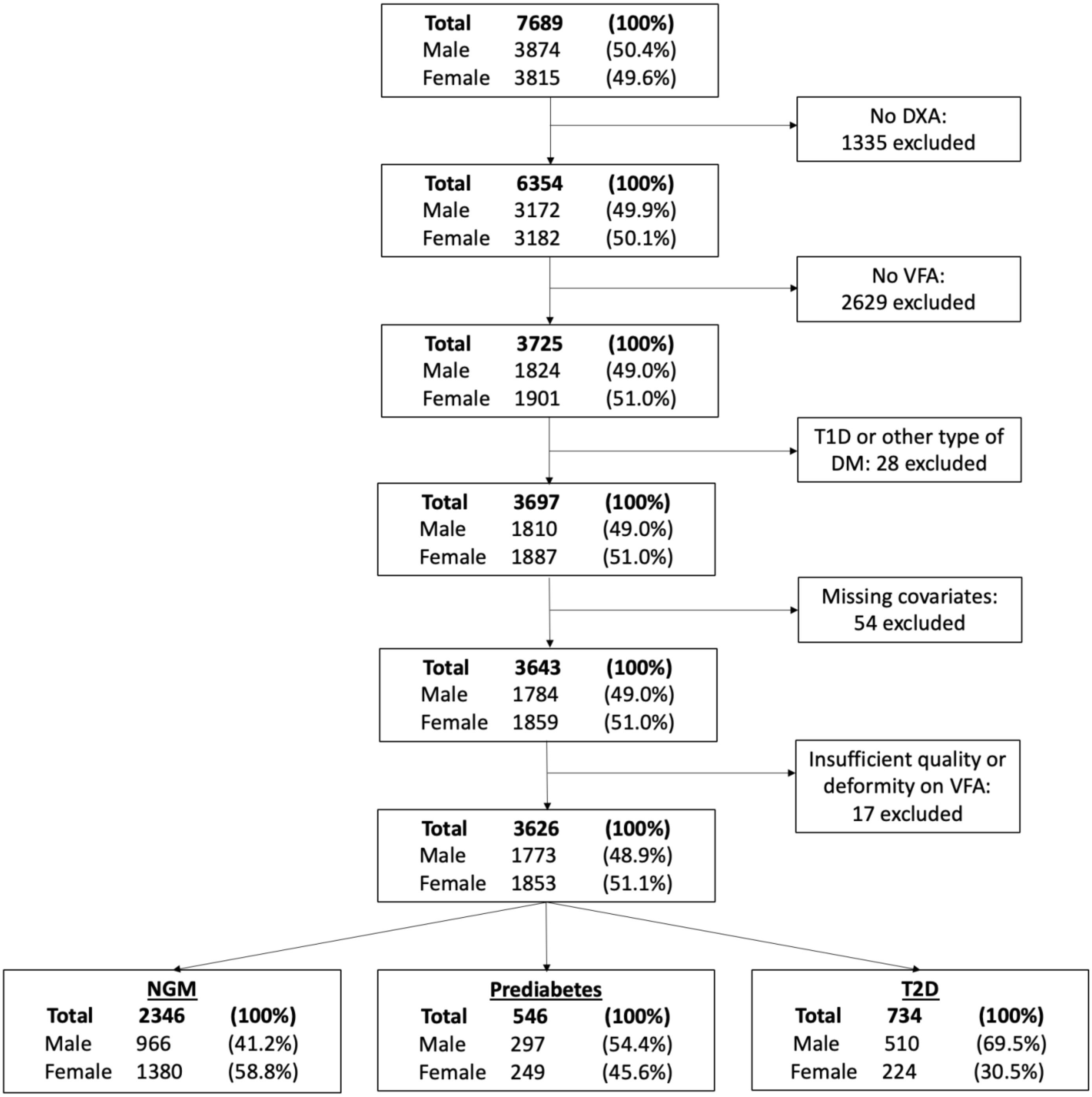

DXA, dual energy X-ray absorptiometry; VFA, vertebral fracture assessment; T1D, type 1 diabetes; DM, diabetes mellitus; NGM, normal glucose metabolism; T2D, type 2 diabetes.
